# Bilayer Membrane Composed of Mineralized Collagen and Chitosan Cast Film Coated With Berberine-Loaded PCL/PVP Electrospun Nanofiber Promotes Bone Regeneration

**DOI:** 10.3389/fbioe.2021.684335

**Published:** 2021-07-19

**Authors:** Yuhan Zhang, Ting Wang, Juan Li, Xiaoming Cui, Mingxia Jiang, Mogen Zhang, Xiaoli Wang, Weifen Zhang, Zhijun Liu

**Affiliations:** ^1^Clinical College, Weifang Medical University, Weifang, China; ^2^Shandong Engineering Research Center for Smart Materials and Regenerative Medicine, Weifang Medical University, Weifang, China; ^3^College of Pharmacy, Weifang Medical University, Weifang, China; ^4^College of Medical Imaging, Weifang Medical University, Weifang, China; ^5^Department of Microbiology, Weifang Medical University, Weifang, China

**Keywords:** berberine, chitosan, bone regeneration, electrospinning, mineralized collagen, nanofiber, cast film

## Abstract

Bone defects are difficult to repair and reconstruct as bone regeneration remains technically challenging, with exogenous factors required to accelerate this process. Biodegradable synthetic scaffolds are promising materials for stimulating bone tissue repair. In this study, we investigated whether a bilayer membrane that includes mineralized collagen (MC) and chitosan (CS) delivering berberine (BER)—a typical Chinese herbal monomer—could promote bone healing in a rat model. An MC/CS cast film was coated with polycaprolactone (PCL)/polyvinylpyrrolidone (PVP) electrospun nanofibers loaded with BER, yielding the BER@PCL/PVP-MC/CS bilayer membrane. The 3-dimensional structure had nanofibers of uniform diameter and showed good hydrophilicity; the bilayer membrane showed favorable mechanical properties. BER@PCL/PVP-MC/CS enhanced the proliferation and attachment of MC3T3-E1 cells *in vitro* and induced bone regeneration when implanted into a rat femoral bone defect. These findings provide evidence that BER@PCL/PVP-MC/CS has clinical potential for effective bone repair.

## Introduction

The function of bone is to protect and support the body. The incidence of bone defects is increasing for various reasons including injury, tumors, infection, and the aging of the global population, resulting in an increase in healthcare costs and decreased work productivity and life quality (Yelin et al., 2016).

Implant materials play an important role in promoting bone regeneration. The main types of implant material used in clinical practice are autologous, allogeneic, and artificial bone. Autologous bone transplantation is considered as the gold standard but is limited by the amount of available tissue, existing defects (mainly morbidity and hematoma at the harvest site), and poor remodeling of the implanted bone (Goulet et al., 1997; Moore et al., 2001; Giannoudis et al., 2005; Laurencin et al., 2006; Dimitriou et al., 2011; Zhang et al., 2014). Allogeneic bone transplantation overcomes these shortcomings but is less osteoinductive and has the risk of immune rejection and infection (Moore et al., 2001; Delloye et al., 2007). In terms of artificial bone implantation, metal and ceramic materials face the problem of non-biodegradability (Gentile et al., 2014; Wang et al., 2015; Yunus Basha et al., 2015; Rh Owen et al., 2018), while biodegradable materials have low osteoinductive capacity (Salgado et al., 2004; Karageorgiou and Kaplan, 2005; LeGeros, 2008).

Electrospinning is a promising technology for tissue engineering. Fibers generated by electrospinning are biodegradable and have high porosity, allowing new bone growth while providing an environment similar to the extracellular matrix (ECM) that promotes cell proliferation and attachment. Polycaprolactone (PCL) is a synthetic polymer that is often used for electrospinning because of its good mechanical properties including elasticity as well as biodegradability and biocompatibility (Singh et al., 2014; Aragon et al., 2017; Tiwari et al., 2017); however, the hydrophobicity and low water absorption of PCL limit its biomedical applications (Singh et al., 2015). These problems can be addressed by combining PCL with water-soluble polymers (Phipps et al., 2012; Tiwari et al., 2017) such as polyvinyl alcohol (Kim et al., 2006), polyethylene oxide (Kim et al., 2007), and polyvinylpyrrolidone (PVP). Nanofibers electrospun from the combination of PCL and PVP have excellent solubility and biocompatibility; PVP was shown to accelerate the biodegradation and increase the hydrophilicity of PCL (Jia et al., 2011; Kim et al., 2013; PranavKumar Shadamarshan et al., 2018). The osteoinductive activity of electrospun nanofibers can be enhanced by the addition of growth factors or natural small molecules. Growth factors are costly to produce and have low stability (Haidar et al., 2009; Awale et al., 2017), and there are unknown risks associated with the application of doses that far exceed physiologic concentrations (Wozney, 1992). Berberine (BER) is a monomer used in Chinese herbal medicine to treat various diseases including bone defects and osteoporosis (Olivares-Navarrete et al., 2012; Tao et al., 2016; Wong et al., 2020; Ma et al., 2021). Recent studies have demonstrated its therapeutic potential in promoting bone regeneration (Martiniakova et al., 2020; Sujitha et al., 2020; Wong et al., 2020).

Mineralized collagen fibers are an essential component of bone tissue that are important for bone regeneration and remodeling. Regenerated bone tissue often shows a poorly mineralized structure (Liu et al., 2019). The mineralization of biomaterials has been widely investigated in bone tissue engineering (Ahmad et al., 2018; Liu et al., 2019; Lee et al., 2020). Undissolved mineralized collagen powder (MC) composed of type I collagen and nanohydroxyapatite has been developed that has a chemical composition and microstructure similar to natural bone (Liao et al., 2003, 2004) and can thus provide a more bionic microenvironment for bone tissue regeneration. MC powder has been approved by the China Food and Drug Administration for clinical applications. Chitosan (CS), the product of chitin deacetylation (Tao et al., 2020), is frequently used as a drug carrier owing to its biodegradability, bioadhesion, low toxicity, and low production cost (Hamedi et al., 2018; Kravanja et al., 2019).

In the present study, for the first time, we designed a cast film composed of MC with CS, which was then coated with a electrospun nanofiber membrane loaded with BER by electrospinning. Cast film technology was chosen to provide a reasonable method of applying MC powder, which is good at bone regeneration but limited by insolubility. The electrospun technology increased the solubility of BER and potentially reduced the first-pass effect (Liu et al., 2010). We evaluated the structural, physicochemical, and mechanical properties of the BER@PCL/PVP-MC/CS bilayer membrane and its ability to promote bone regeneration using *in vitro* and *in vivo* models.

## Materials and Methods

### Materials

PCL and PVP, the biodegradable polymers used in electrospinning, were purchased from Shanghai Yuanye Bio-Technology Co. (Shanghai, China). MC powder was from Beijing Aojing Medical Instruments Co. (Beijing, China). CS (12–16 kDa) was from Shandong Aokang Bio-Technology Co. (Dongying, China). Trichloromethane, methyl alcohol, and glacial acetic acid were from Yantai Far Eastern Fine Chemical Co. (Yantai, China). BER was from Macklin Biotechnology Co. (Shanghai, China). The mouse osteoblast cell line MC3T3-E1 (Subclone 14) and α-Minimal Essential Medium used in cell experiments were from Procell Life Science and Technology Co. (Wuhan, China). Fetal bovine serum was from Zhejiang Tianhang Biotechnology Co. (Huzhou, China). Phosphate-buffered saline, penicillin, streptomycin, and 3-(4,5-dimethyl-2-thiazolyl)-2,5-diphenyl-2-H-tetrazolium bromide (MTT) were from Beijing Solarbio Science and Technology Co. (Beijing, China). The Hoechst Staining Kit was from Beyotime Biotechnology Co. (Shanghai, China). The fluorescein isothiocyanate (FITC) Phalloidin Staining Kit was from Shanghai Yeasen Biotechnology Co. (Shanghai, China).

### Preparation of BER@PCL/PVP-MC/CS Bilayer Membrane

#### Preparation of MC/CS Cast Film

**FIGURE 1:**
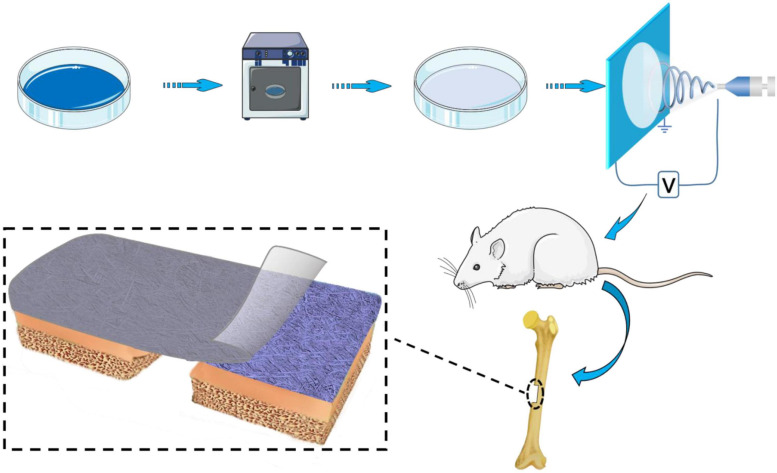
Schematic illustration of the method of preparation and implantation of the BER@PCL/PVP-MC/CS bilayer membrane.

The method of preparation of BER@PCL/PVP-MC/CS is shown in Figure 1. The cast film was made from CS (12–16 kDa) and MC. Acid-swollen collagen (0.67 g/l) was mixed with calcium and phosphate ion solution (Ca/P = 1.67) and the pH was adjusted with NaOH. After allowing the solution to stand for 48 h at room temperature, the supernatant was removed and the sediment was washed and dried to obtain MC powder (Zhang et al., 2003). CS was prepared as a 2% (w/v) solution in 1% (v/v) glacial acetic acid, and 0.8 wt% MC was added to the solution while stirring. A 5-mL volume of the solution was added to a Petri dish and dried in an oven at 37°C for 150 min. The cast film was carefully removed with tweezers.

#### Preparation of BER@PCL/PVP-MC/CS

PCL/PVP electrospun nanofibers were prepared by combining 10% (w/v) PCL and 30% (w/v) PVP in a chloroform:methanol (3:1) solvent. The optimal volume ratio of PCL:PVP to obtain fibers was 70:30. BER was added to the PCL/PVP solution at 3 different concentrations (1, 10, and 100 μM) with thorough mixing. The prepared electrospinning solution was loaded into the injection device using a 5-mL syringe. The electrospinning conditions were as follows: Push rate, 0.05 mm/min; distance from the needle to the collection device, 13 cm; and working voltage, 13 kV. MC/CS cast film was coated with BER-loaded nanofibers (10 mL), yielding a BER@PCL/PVP-MC/CS bilayer membrane that was stored in a vacuum dryer.

### Scanning Electron Microscopy Analysis of BER@PCL/PVP-MC/CS Morphology

The surface morphology of BER@PCL/PVP electrospun nanofibers and control nanofibers was characterized using a field emission scanning electron microscope (MERLIN; Carl Zeiss, Oberkochen, Germany); fiber diameter was measured using Nano Measurer software^1^.

### Fourier Transform Infrared Spectroscopy (FT-IR)

We confirmed that BER was loaded onto the electrospun nanofibers and did not interact with other components by FT-IR spectrometry (Avater-360; PerkinElmer, Waltham, MA, United States).

### Differential Scanning Calorimetry (DSC) and Thermogravimetric Analysis (TGA)

The thermal behavior of the BER@PCL/PVP-MC/CS bilayer membrane and its constituents was evaluated by DSC and TGA using a simultaneous thermal analysis system (Sinmo, Frankfurt, Germany). TGA was used to evaluate thermal stability, with weight changes recorded at a heating rate of 2°C/min within the range of 25–900°C.

### Contact Angles

Contact angles were measured at room temperature after 10-s dropwise addition of water onto the material surface with a goniometer (Kruss, Hamburg, Germany).

### Mechanical Characterization

Tensile testing was performed on a universal testing machine (AG2000A; Shimadzu, Kyoto, Japan). Specimens were tested at a constant displacement rate of 3 mm/min.

### *In vitro* Release of BER@PCL/PVP

The drug release profiles of the BER@PCL/PVP electrospinning nanofibers were determined by immersing the samples in PBS (pH 7.4). Each membrane was cut into 500 mg and transferred to a dialysis bag with 5 mL PBS, the dialysis bag was dispersed in 45 mL PBS and placed in 37°C under shaking at 100 rpm (*n* = 3). According to scheduled time intervals (from 15 min to 28 days), 1 mL PBS was collected and replaced with an equal volume of fresh PBS, then stored at–20°C until analysis.

The amount of released drug was detected by high performance liquid chromatography (HPLC, Thermo UltiMate 3000, Germany). The column used for separation of the berberine was an ELITE ODS, C18, 5 μm, 4.6 mm × 250 mm HPLC column. The mobile phase contained 0.05 mol/L KH_2_PO_4_ and acetonitrile (75/25, v/v). The absorbency was monitored at 345 nm and the flow rate was 1.0 mL/min. The drug release was calculated according to the initial weight of the drug incorporated in the electrospinning nanofibers.

### Proliferation of MC3T3-E1 Cells *in vitro*

The biocompatibility of the BER@PCL/PVP-MC/CS bilayer membrane was assessed and the optimal concentration of BER was determined by MTT assay using MC3T3-E1 cells. The cells were seeded in 96-well plates at 2 × 10^3^/well and incubated at 37°C and 5% CO_2_ for 24 h. A 0.15-g membrane sample was UV-sterilized and added to 1 mL of culture medium. The leachate was filtered and added to the wells after removing the culture medium. The cells were divided into 3 groups (*n* = 5 wells per group): (1) control (cells cultured in complete medium); (2) BER sample (cells cultured in complete medium containing 10 μM BER); and (3) leachate sample (cells cultured on 4 BER@PCL/PVP-MC/CS samples). Cell proliferation was evaluated on day 1, 3, 5, and 7. A 10-μL volume of MTT (5 mg/mL) was added to each well followed by incubation for 4 h; after removing the supernatant, 100 μL of dimethylsulfoxide was added to each well to dissolve the formazan crystals, and the absorbance at 490 nm was measured with a microplate reader.

### Adherence of MC3T3-E1 Cells *in vitro*

The morphology of MC3T3-E1 cells attached to the BER@PCL/PVP-MC/CS bilayer membrane was observed by confocal laser scanning microscopy (CLSM, TCS SP8; Leica, Wetzlar, Germany). The cells were divided into 2 groups: (1) MC cast film (cells cultured on cast film); and (2) BER@PCL/PVP-MC/CS (cells cultured on 4 BER@PCL/PVP-MC/CS samples). Cells were seeded in 6-well plates at 2 × 10^5^/well and incubated at 37°C and 5% CO_2_ for 6 and 24 h. After incubation, the cells were fixed with 4% paraformaldehyde at room temperature, then cell nuclei were stained by Hoechst 33258 and the cytoskeleton were stained with Alexa Fluor 488 phalloidin (AF 488) under shading at 37°C.

### Animals and Surgical Procedure

All experimental procedures were performed according to the guidelines for laboratory animals established by the Animal Care and Use Committee of Weifang Medical University. Male Sprague-Dawley rats (6–8 weeks old and weighing 210–220 g) (Vital River, Beijing, China) were anesthetized by intraperitoneal injection of 3% pentobarbital solution. Under strict aseptic conditions, a membrane was implanted into the right femur. Briefly, the surgical areas were shaved, then sterilized by iodine and 70% ethanol before surgery. To establish the model of femoral defect in SD rats, the femur was exposed by an incision through the skin, fascia, and periosteum layer by layer. A bone defect of 5 mm × 3 mm which is deep into the marrow cavity was made on the shaft of the femur near the femoral head. The membrane was placed in the face of the bone defect (*n* = 5 rats per group). The groups were as follows: (1) control (rats without a defect); (2) model (rats with a defect but without treatment); (3) treatment (rats with a defect that were treated with different membranes: BER@PCL/PVP electrospun nanofibers; MC/CS cast film; BER@PCL/PVP-MC/CS bilayer membrane; or a commercial MC membrane fabricated by Beijing Aojing Medical Instruments Co. (Beijing, China). At the end of the surgery, the tissues were sutured in layers. The animals were allowed to heal for 4 or 8 weeks and then sacrificed, and the femur and organs were harvested for analysis.

### *In vivo* Evaluation of Biocompatibility

#### Hematologic Assessment

A blood sample was collected from each rat by cardiac puncture at the time of sacrifice (4 and 8 weeks postsurgery) for routine blood examination. Erythrocytes, hemoglobin, platelets, and 5 types of leukocyte were quantified.

#### Histologic Analysis

Gathered viscus tissues (heart, liver, spleen, lung, and kidney) of rats at 4 and 8 weeks, postsurgery fixed in 4% formaldehyde, paraffin embed. The samples were deparaffinized and rehydrated, then sections were cut and stained with hematoxylin and eosin (H&E), morphology observed by microscope to evaluate the biocompatibility of the different groups.

### *In vivo* Evaluation of Tissue Regeneration

#### Imaging

Femur samples were collected from rats and scanned by X-ray computed tomography (CT) (Optima CT 670; General Electric, Boston, MA, United States). 3-Dimensional (3D) images were reconstructed using RadiAnt DICOM Viewer software (Medixant, Poznań, Poland) to evaluate bone healing.

#### Histology

Femur samples were fixed and decalcified and cut into thin sections (Liao et al., 2004). Some were stained with H&E as described in **section** 2.12.2, and others were deparaffinized and rehydrated for Goldner’s trichrome staining. The sections were observed with a light microscope.

### Statistical Analysis

SPSS v22.0 (SPSS Inc., Chicago, IL, United States) was used to analyze data, which are presented as the mean ± standard deviation of at least 3 independent experiments. The Student’s *t*-test and one-way analysis were used to determine the statistical significance. With *P* < 0.05 was considered as statistically significant.

## Results and Discussion

### Analysis of Surface Morphology

**FIGURE 2:**
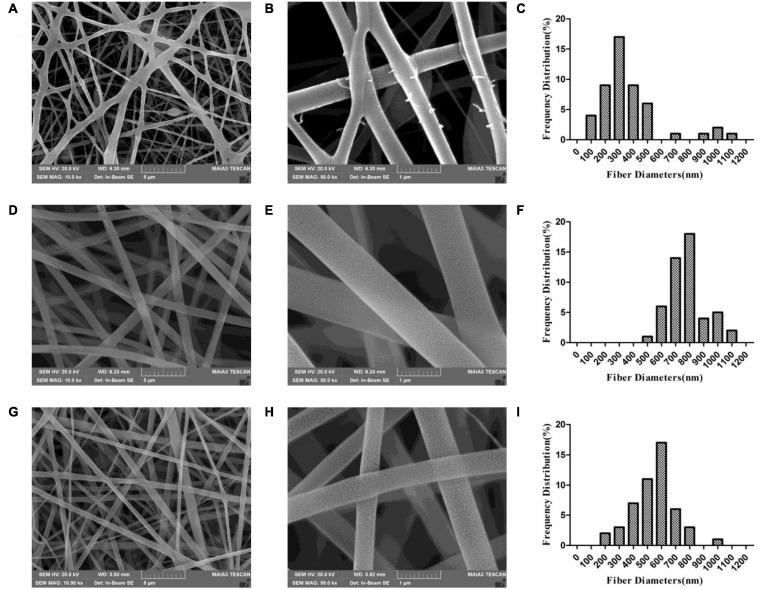
**(A–I)** Scanning electron micrographs of PP-21G **(A,B)**, PP-20G **(D,E)**, and PP-BER-20G **(G,H)** scaffolds at low **(A,D,G)**, high **(B,E,H)** magnification, and distribution of nanofiber diameters **(C,F,I)**.

Scanning electron microscopy analysis revealed that the electrospun membrane consisted of interconnected randomly oriented fibers in a 3D structure with high porosity (Figure 2). We examined whether there were differences between nanofibers spun with different needles under the same conditions. With a 21-G needle, there were beads in the fiber; the fibers were adherent and had variable diameter, with an average value of 364 ± 222 nm. With a 20-G needle, the diameter of the nanofibers was uniform (780 ± 124 nm on average) and there was no adhesion, but cracks were observed at high magnification that increased the roughness of the stent. The scaffold generated by electrospinning had a structure similar to natural ECM (Politi et al., 2020); the porosity can ensure the flow of nutrients, oxygen, and metabolic waste products from cells and accommodate cell growth and migration, which is important for tissue regeneration (Li et al., 2019). Meanwhile, the mimic of physiological microenvironment can accelerate the bone regeneration (Moradi et al., 2018). For subsequent experiments we selected fibers spun with a 20-G needle. The surface roughness of the fibers was further increased after loading BER. The average diameter of nanofibers in the BER@PCL/PVP-MC/CS membrane was 548 ± 150 nm.

### FT-IR Analysis

**FIGURE 3:**
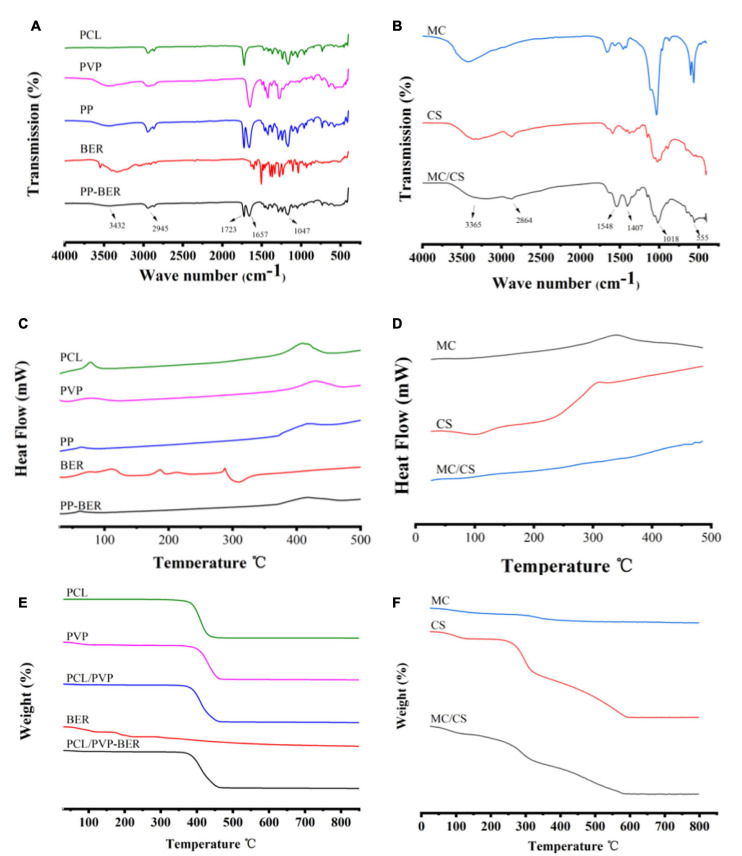
**(A,B)** FT-IR spectra of PCL, PVP, PCL/PVP electrospun nanofibers (PP), BER, and BER@PCL/PVP electrospun nanofibers (PP-BER) **(A)**; and MC, CS, and MC/CS cast film **(B)**. **(C,D)**. DSC curves of PCL, PVP, PP, BER, and PP-BER **(C)**; and MC, CS, and MC/CS cast film **(D)**. **(E,F)**. TGA curves of PCL, PVP, PP, BER, and PP-BER **(E)**; and MC, CS, and MC/CS cast film **(F)**.

The IR spectrum of PCL showed characteristic peaks at 2943.60 cm^–1^ (-CH_2_-), 1721.84 cm^–1^ (–C = O), and 1107.72 cm^–1^ (-C-O-C-) (Figure 3A; Ranjbarvan et al., 2017); the spectrum for PVP showed peaks at 2907.72 cm^–1^ (-CH_2_-) and 1649.70 cm^–1^ (–C = O); and the BER spectrum showed peaks at 1035.14 cm^–1^ (-C-O-C-) and at 1599.69, 1566.19, and 1458.07 cm^–1^ (benzene ring) (Jin et al., 2020). In the spectrum of the bilayer membrane, there were peaks at 1,723 cm^–1^ (PCL), 1,657 cm^–1^ (PVP), and 1,047 and 3,432 cm^–1^ (BER), in addition to the –CH_2_- peak at 2,945 cm^–1^ ascribed to PCL and PVP. These results demonstrate that no chemical interactions occurred between BER and the carrier. The IR spectra of MC, CS, and MC/CS cast film are shown in Figure 3B. Peaks at 1,449 and 561 cm^–1^ were observed for MC (Huang C., 2016). The spectrum of CS showed peaks at 3,365 cm^–1^ (–OH and –NH), 2,866 cm^–1^ (–CH_2_–), 1,580 cm^–1^ (–NH_2_), and 1,026 cm^–1^ (amide III) (Karpuraranjith and Thambidurai, 2017). The peaks of the cast film at 3,328, 2,862, 1,403, 1,022, and 555 cm^–1^ were consistent with those observed for the raw materials, indicating that no chemical reaction had occurred during film preparation.

### DSC and TGA

The physical state of BER in the nanofibers was investigated by DSC. The DSC curves of PCL, PVP, PCL/PVP nanofibers, BER, and BER-loaded nanofibers are shown in Figure 3C. The DSC thermogram of BER showed a sharp endothermic peak at 190°C, assigned to its melting point (Zhang et al., 2013), and another sharp melting endotherm at 287°C, which are absent in the thermogram of the electrospun nanofibers, indicating that the BER existed in an amorphous form. The TGA curves showed that BER@PCL/PVP had a higher decomposition temperature than its constituents at around 370°C (Figure 3E), suggesting that the membrane was more stable.

As for the cast film, CS had a prominent exothermic peak at around 220–320°C (Figure 3D), corresponding to the weight loss observed in the TGA (Figure 3F) and indicating that at this temperature, CS was decomposed by and released heat. MC had an exothermic peak at 341°C, corresponding to the weight loss in the TGA spectrum at this temperature. The cast film’s DSC curve had a similar shape but there was no obvious exothermic peak, only a trend of slow heat loss. The TGA spectrum of the cast film showed significant heat loss at 180°C that continued to 340°C, corresponding to the changes caused by MC and CS and indicating a higher content of the latter.

### Contact Angle

**FIGURE 4:**
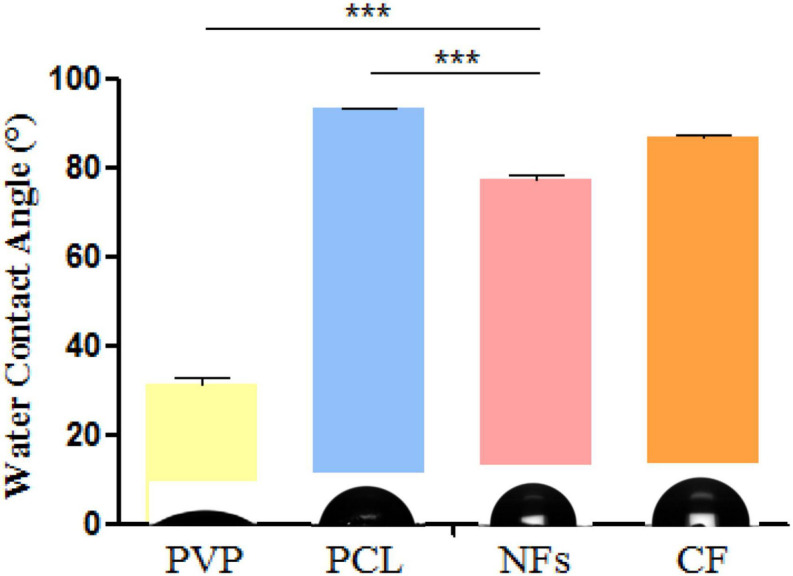
Water contact angle measurements of electrospun nanofiber membrane and its component materials PCL and PVP. *P < 0.05, **P < 0.01, ***P < 0.001.

The contact angle measures the degree of hydrophilicity, which influences cell adhesion and proliferation (Jia et al., 2011). We measured the contact angles of PCL, PVP, and the electrospun nanofiber membrane and found that after adding PVP, the contact angle of nanofibers (77.53°) was decreased compared to that of PCL (93.16°) (Figure 4), which is in agreement with a previous report (Jia et al., 2011). Meanwhile, it was found that the contact angle of CF membrane (86.80°) was close to that of nanofibers, but the hydrophilicity was not as good as that of nanofibers.

### Mechanical Properties

**FIGURE 5:**
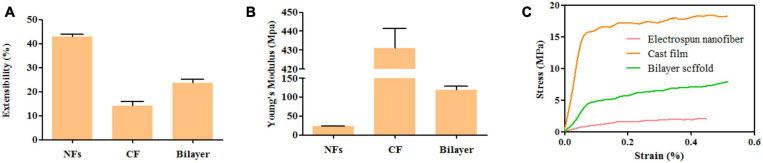
**(A–C)**. Extensibility **(A)**, Young’s modulus **(B)**, and tensile stress–strain curves **(C)** of nanofiber, cast film, and bilayer membrane.

The mechanical properties of the membrane were investigated by evaluating the elongation at break (Figure 5A), Young’s modulus (Figure 5B), and stress–strain relationship (Figure 5C). The adding of BER@PCL/PVP affects the elasticity of specimens, Figures 5A,B showed that the bilayer membrane has an increase in elongation at break and decrease in Young’s modulus by the adding of BER@PCL/PVP to MC/CS cast film (Yahia et al., 2019). As apparent from the Figure 5C, MC/CS cast film needs more force to produce the same degree of deformation, indicating that MC/CS cast film has good mechanical support under elongation, thus the bilayer membrane showed improved mechanical strength under elongation by combine the MC/CS cast film with BER@PCL/PVP electrospun nanofibers.

### *In vitro* Release

The drug release profiles of the membranes were shown in Supplementary Figure 1. The results showed that more than 30% BER had been burst released from electrospinning nanofibers in the first day, especially at the 8th h, may be related to the good water solubility of PVP; then, an approximately linear release of BER was observed from 2nd to the 7th day. The remaining BER had a relatively slow and continuous release till the 28th day, which reached about 65%. Similar trend about BMP2 was considered as ideal release strategy, available research indicated that the initial burst helps to recruit osteoprogenitor cells and the subsequent sustained release promotes osteogenic differentiation (Lee and Koh, 2014).

### Effect of the Bilayer Membrane on MC3T3-E1 Cell Proliferation and Attachment *in vitro*

**FIGURE 6:**
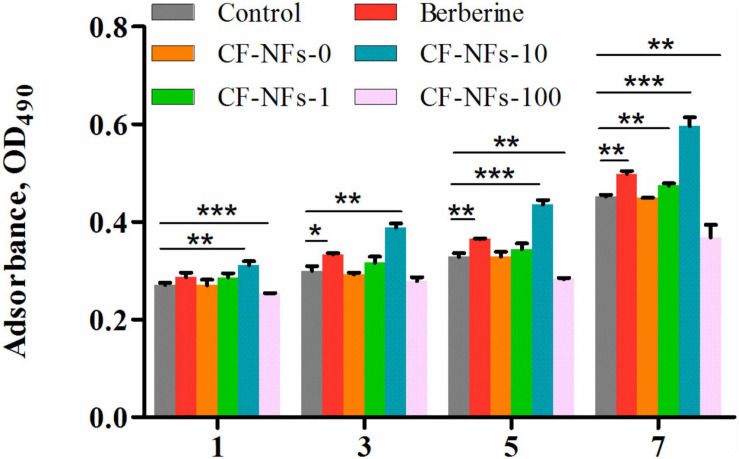
Evaluation of cell proliferation with the MTT on day 1, 3, 5, and 7 of culture. *P < 0.05, **P < 0.01, ***P < 0.001.

**FIGURE 7:**
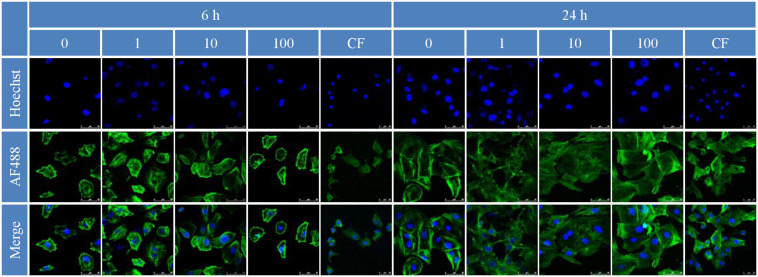
Cell attachment 6 and 24 h after inoculation observed by confocal laser confocal microscopy.

The *in vitro* proliferation of MC3T3-E1 cell on bilayer membrane was assessed by MTT assay to demonstrate the cells count after 1, 3, 5, and 7 days (Son et al., 2013). The results of the MTT assay showed that a BER concentration in membrane of 10 μmol/l was optimal for promoting MC3T3-E1 cell proliferation (*P* < 0.05) (Figure 6). As for attachment, the effect of membranes on cell cytoskeletal morphology after 6 and 24 h culture was revealed using CLSM. At 6 h after seeding on the various samples, the cells showed no differences in morphology among all the groups, with a spherical shape and minimal spreading (Figure 7). After 24 h, cells grown on BER@PCL/PVP-MC/CS had a polygonal shape and showed greater spreading than those

grown on MC/CS cast film, which had a narrow form and the filopodia of cells extended along random nanofibers in multiple directions (Qian et al., 2019).

### Biocompatibility

**FIGURE 8:**
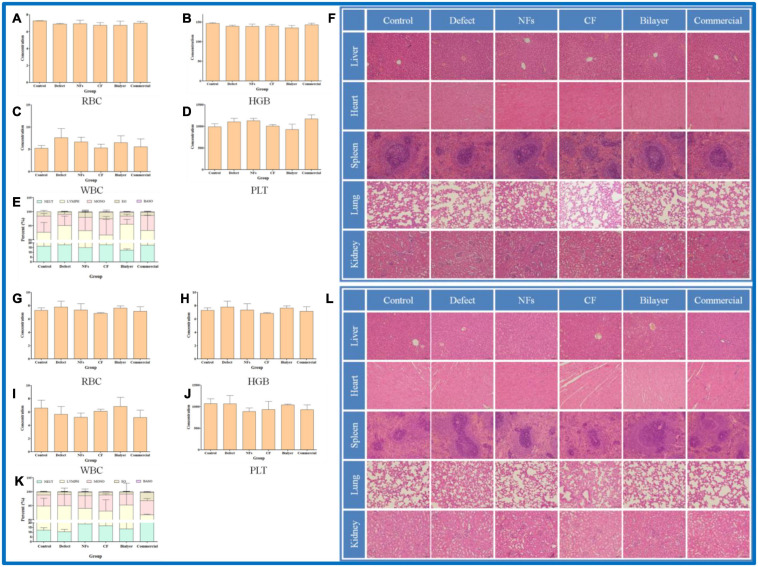
**(A–L)** Biocompatibility evaluation of membranes *in vivo* at 4 weeks **(A–F)** and 8 weeks **(G–L)**. Percentage of red blood cells **(A,G)**, hemoglobin **(B,H)**, leukocytes **(C,I)**, and platelets **(D,J)** and 5 types of leukocyte **(E,K)** in rats. **(F,L)** Histopathologic analysis by H&E staining of liver, heart, spleen, lung, and kidney tissues. Scale bar, 100 μm.

To evaluate the toxicity of BER@PCL/PVP-MC/CS, blood samples collected from rats 4 and 8 weeks postsurgery were analyzed (Murabayashi and Nose, 2013). The number of erythrocytes, hemoglobin, platelets, and leukocytes were all within the normal ranges and comparable to the numbers in the untreated controls (Figures 8A–E,G–K). H&E staining of organ tissue harvested at the same time points revealed no obvious signs of inflammation or pathology in rats implanted with BER@PCL/PVP-MC/CS (Figures 8F,L). These results demonstrate that the bilayer membrane has good biocompatibility.

### Bone Regeneration *in vivo*

**FIGURE 9:**
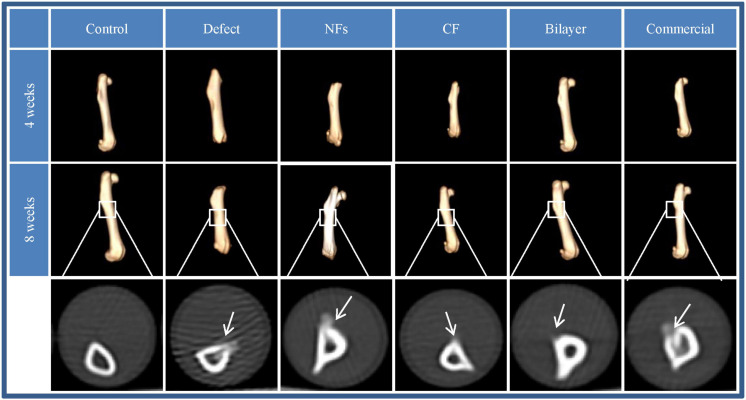
Images obtained by 3D CT of bone regeneration at the bone–implant interface in rats at 4 and 8 weeks.

**FIGURE 10:**
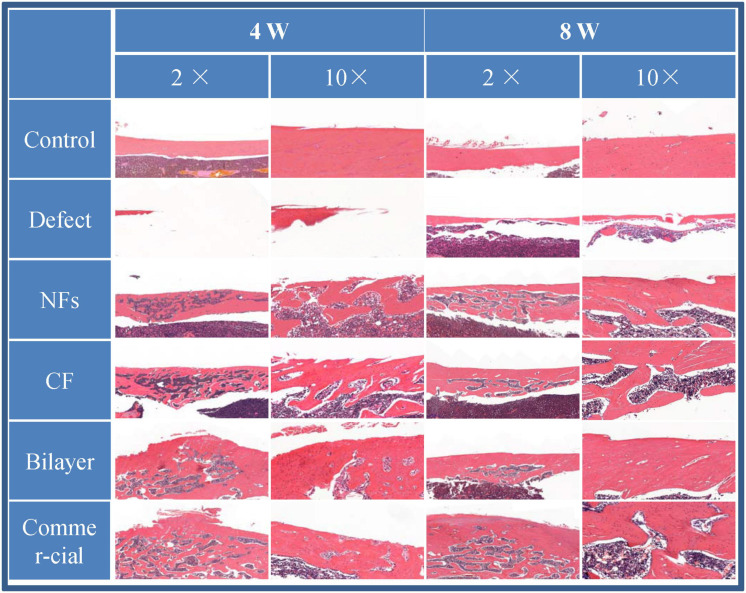
H&E staining of the bone defect after 4 and 8 weeks of implantation.

**FIGURE 11:**
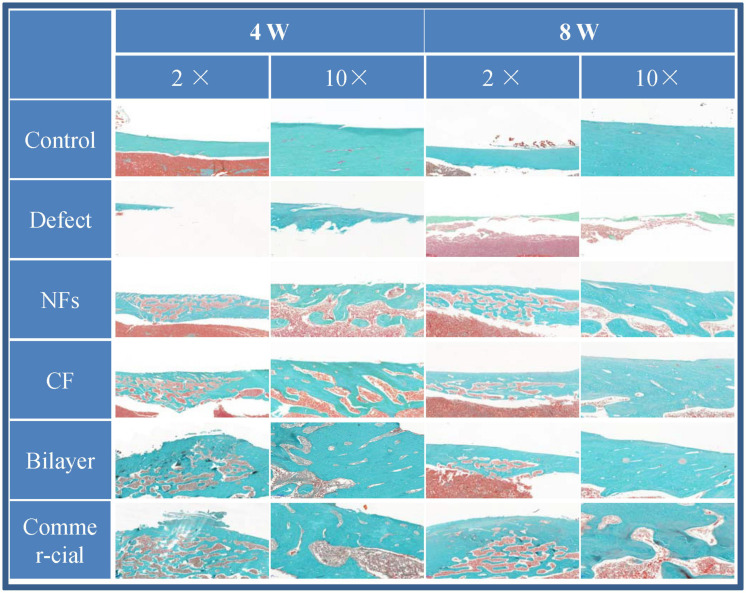
Goldner’s trichrome staining of the bone defect after 4 and 8 weeks of implantation.

The effect of BER@PCL/PVP-MC/CS on bone regeneration *in vivo* was evaluated by CT and histologic examination. The CT scan revealed that the bilayer membrane and commercial membrane achieved the best results in terms of promoting bone healing and were comparable in their efficacy (Figure 9), although rats implanted with the single membrane (electrospun nanofiber or cast film membrane) showed some improvement at week 8 compared to week 4. Meanwhile, rats with an untreated defect showed poor bone healing and non-union. H&E and Goldner’s trichrome (Imagawa et al., 2020) staining revealed that at 4 weeks postsurgery, repair of the bone defect occurred slowly in the defect group, as evidenced by discontinuity in the bone cortex, non-union of bone, and an absence of tissue and cells (Figures 10, 11). At low magnification, a small amount of regenerated bone was observed around the defect edges. In contrast, rats that were implanted with the biomaterials had thick and loosely braided newly formed bone, abundant vasculature, and more cell growth compared to untreated rats. Consistent with the results of the CT scan, rats with the BER@PCL/PVP-MC/CS and commercially manufactured implants showed the greatest improvement in bone defect repair: A thin layer of dense lamellar bone was formed in the same direction as the applied force. At 8 weeks postsurgery, near-continuous bone tissue was observed in the model group, but the tissue was relatively thin, with sparse blood vessels and few cells. More woven bone was present in treatment groups along with more lamellar bone in the cortical area. In the BER@PCL/PVP-MC/CS group there was thicker lamellar bone with higher bone density, with only a small amount of braided bone remaining.

## Conclusion

In this study, we developed a bilayer membrane with good bioactivity and mechanical characteristics by coating a MC/CS film with BER-loaded PCL/PVP, provides a reasonable solution for diversified addition in surgery. The electrospun nanofibers had a structure similar to ECM that induced the attachment and proliferation of osteoblasts *in vitro* and can potentially enhance the cell–cell interactions and cell migration necessary for bone healing. This study also brings forward a method of applying insoluble MC powder, which provides a reasonable solution for the problem of difficult addition in surgery. *In vivo* experiments demonstrated that the combination of BER and MC for the first time stimulated bone tissue repair when implanted into a femoral bone defect in adult rats for 4 and 8 weeks. In conclusion, the developed membranes with bilayer structures are a promising solution for bone regeneration.

## Data Availability Statement

The original contributions presented in the study are included in the article/Supplementary Material, further inquiries can be directed to the corresponding author.

## Ethics Statement

The animal study was reviewed and approved by the Animal Ethics Committee of Weifang Medical University.

## Author Contributions

All authors listed have made a substantial, direct and intellectual contribution to the work, and approved it for publication.

## Conflict of Interest

The authors declare that the research was conducted in the absence of any commercial or financial relationships that could be construed as a potential conflict of interest.

## Footnotes

1 https://nano-measurer.software.informer.com/1.2/

## Supplementary Material

Supplementary Figure 1 *In vitro* release study.
