# Supplementary figures and images for: Bilayer Membrane Composed of Mineralized Collagen and Chitosan Cast Film Coated With Berberine-Loaded PCL/PVP Electrospun Nanofiber Promotes Bone Regeneration
Source: Front Bioeng Biotechnol. 2021 Jul 19;9:684335. doi: 10.3389/fbioe.2021.684335 (PMC8327095; doi:10.3389/fbioe.2021.684335)

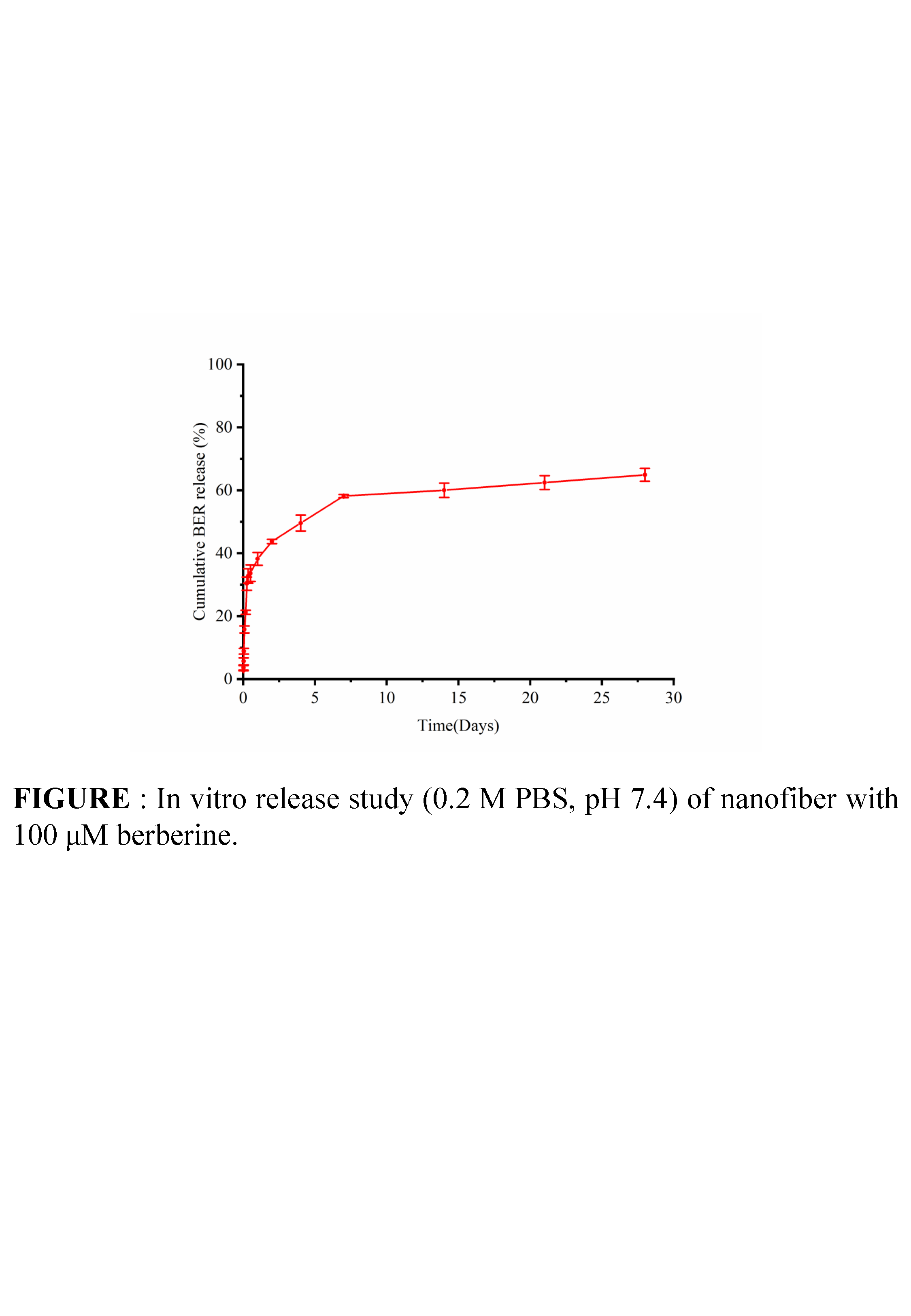

Supplement: Supplementary Figure 1 — In vitro release study. [file Image_1.tiff]
